# Literary Notes

**Published:** 1896-07

**Authors:** 


### Literary Notes.

THE *Medical Record*, issued June 13, 1896, is liberally devoted to the consideration of summer health resorts, and is adequately named a Summer Health Resort Number. It is considerably increased in size and has an illuminated cover in antique laid paper typical of this special edition. In addition, it contains a number of illustrated advertisements of the principal summer health resorts, and is altogether the handsomest specimen of weekly medical magazine art that we have seen in many a day.

A CURIOUS decision relating to American degrees is reported in England. Justices Grantham and Collins, eminent judges of the high court of justice of England, decided in a case of the Medical Defense Union vs. Bridgewater that having invariably added the letters U. S. A. after the title M. D. there was no intent to defraud, hence no cause of action at law. The Union is mulcted in about $4,000 costs. It appears that Bridgewater is an American physician holding diplomas from New York and Philadelphia medical colleges. This decision puts a new color on the American diploma question.

